# Atrial Tachyarrhythmia in Rgs5-Null Mice

**DOI:** 10.1371/journal.pone.0046856

**Published:** 2012-11-05

**Authors:** Mu Qin, He Huang, Teng Wang, He Hu, Yu Liu, Yongwei Gu, Hong Cao, Hongliang Li, Congxin Huang

**Affiliations:** 1 Department of Cardiology, Renmin Hospital of Wuhan University, Wuhan, China; 2 Cardiovascular Research Institute, Wuhan University, Wuhan, China; Temple University, United States of America

## Abstract

**Aims:**

The aim of this study was to elucidate the effects of regulator of G-protein signaling 5 (Rgs5), a negative regulator of G protein-mediated signaling, on atrial repolarization and tachyarrhythmia (ATA) in mice.

**Methods and Results:**

In present study, the incidence of ATA were increased in Rgs5^−/−^ Langendorff-perfused mouse hearts during program electrical stimulation (PES) (46.7%, 7 of 15) and burst pacing (26.7%, 4 of 15) compared with wild-type (WT) mice (PES: 7.1%,1 of 14; burst:7.1%,1 of 14) (P<0.05). And the duration of ATA also shown longer in Rgs5^−/−^ heart than that in WT, 2 out of 15 hearts exhibited sustained ATA (>30 s) but none of them observed in WT mice. Atrial prolonged repolarization was observed in Rgs5^−/−^ hearts including widened P wave in surface ECG recording, increased action potential duration (APD) and atrial effective refractory periods (AERP), all of them showed significant difference with WT mice (P<0.05). At the cellular level, whole-cell patch clamp recorded markedly decreased densities of repolarizing K^+^ currents including I_Kur_ (at +60 mV: 14.0±2.2 pF/pA) and I_to_ (at +60 mV: 16.7±1.3 pA/pF) in Rgs5^−/−^ atrial cardiomyocytes, compared to those of WT mice (at +60 mV I_to_: 20.4±2.0 pA/pF; I_kur_: 17.9±2.0 pF/pA) (P<0.05).

**Conclusion:**

These results suggest that Rgs5 is an important regulator of arrhythmogenesis in the mouse atrium and that the enhanced susceptibility to atrial tachyarrhythmias in Rgs5^−/−^ mice may contribute to abnormalities of atrial repolarization.

## Introduction

Atrial tachyarrhythmia (ATA), characterized by abnormal, disorganized and rapid atrial electrical activity, is a major public health problem. In particular, atrial fibrillation, which affects 0.5% of people over the age of 50 and 10% of people over 80 [2], is a significant burden on health care resources and increases the risk of serious complications including embolic disease, heart failure and sudden cardiac death. Despite the development of diverse animal models and the application of a multitude of pharmacological and non-pharmacological therapies, understanding of the mechanism underlying ATA remained incomplete. The recent discovery of DNA variants associated with ATA provides new insights into the mechanisms underlying arrhythmias. Changes in the expression of multiple genes including KCNQ1, KCNE1 and KCNE2 have been shown predisposed to atrial fibrillation [1], [4]. Thus, the upstream molecular-regulated targets of pro-arrhythmic substrate need to be better understood.

Rgs5 is a GTPase-activating protein (GAP) for Gα subunits and has an important role in the negative regulation of G-protein-coupled receptor (GPCR)-mediated signaling. Several studies have demonstrated that Rgs5 interacts with Gα(q) and Gα(i) in cardiovascular tissue and inhibits those acting via special GPCRs including AngII type 1 receptor (AT1R) and endothilin-1 receptors [5]. These GPCRs have been implicated in the development of cardiac remodeling during AF, and induced left atrial remodeling and fibrosis are dependent on the extracellular signal-regulated kinase (ERK) pathway [7]. Our previous work showed that Rgs5^−/−^ mice promoted cardiac hypertrophy and fibrosis due to the activation of the MEK-ERK1/2 signaling pathway [15]. As an important regulator of atrial GPCR-dependent signaling, RGS5 protein may potentially exhibit negative way in pro-arrhythmic substrate.

We report here that mice with a knockout of Rgs5 displayed susceptibility of ATA by electrical stimuli and had associated repolarization abnormalities. These observations may provide a new insight into understanding the molecular pathways underlying atrial arrhythmogenesis and for developing strategies for the treatment of atrial tachyarrhythmia.

## Materials and Methods

### 1. Mouse experiments

All animal procedures were performed in accordance with the Guide for the Care and Use of Laboratory Animals, published by the US National Institutes of Health (NIH Publication No. 85-23, revised 1996) and were approved by the Institutional Animal Care and Use Committee at Renmin Hospital of Wuhan University, China.

The generation and genotyping of Rgs5^−/−^ mice (C57BL/6 background) have been described previously. Mice were provided with food and water and held on standard 12 hours light and dark cycles in temperature and humidity controlled house. Male Wild-type and Rgs5^−/−^ mice aged 8 to 10 weeks were used in the studies.

### 2. ECG recordings

Telemetry ECG (Lead II) was continuously recorded in consciously moving mice by Telemetry ECG (DSI, US). The P-wave duration and amplitude (P_dur_ and P_amp_), PR interval and QRS duration was measured.

### 3. Echocardiography and histological analysis

Echocardiography was performed to assess left atrial diameter (LAD), left ventricular end-diastolic diameter (LVEDD), left ventricular end-systolic diameter (LVESD), ejection fraction (EF) and fractional shortening (FS). For histological analysis, hearts were excised and cut transversely close to the apex to visualize the left and right ventricles and atrial appendages. Sections of heart (4–5 µm thick) were prepared and stained with Picro Sirius Red (PSR) for collagen deposition [15].

### 4. Preparation of Langendorff-perfused hearts

The isolated heart was perfused with HEPES-buffered Tyrode's solution. Perfusion was commenced in a retrograde manner through the aorta at 2–2.5 ml/min. The hearts that did not recover to the regular spontaneous rhythm or had inreversible myocardial ischemia were discarded.

### 5. MAP and BEG recording

To examine the atrial electrical activity, the bipolar electrogram (BEG) was recorded from the epicardial surface of atrial appendage using a silver chloride (2 mm tip diameter) recording electrode. The paired platinum stimulating electrodes paced the epicardial surface of right atrial appendage and the stimulation used a 1 ms square-wave stimuli at three times excitation threshold.

### 6. Electrical stimulated protocol

The Programmed electrical stimulation (PES) protocol was used for atrial effective refractory period (AERP) and atrial-ventricular effective refractory period (AVERP) examinations. Sinus node recovery time (SNRT) was measured after a 2 s pacing train at a cycle length of 100 ms [16]. Inducibility of atrial tachyarrhythmia was tested by using both PES and burst pacing (2 ms pulses at 50 Hz, 2 s burst duration) [24], burst pacing used up to 3 minutes of pacing in both atrial locations.

### 7. Transmembrane action potential (TAP) recording

The samples of atrial myocardium were removed from heart and superfused with HEPES-buffered Tyrode's solution. TAP were recorded with borosilicate glass microelectrodes during regular pacing frequency of 1 Hz, 2 Hz, 3.3 Hz, 5 Hz and 6.7 Hz.

### 8. Isolation of atrial cardiac myocytes

Atrial myocytes were isolated by four steps perfused digestion. At the end of the perfusion, the whole ventricle was dissected from the heart, and the atria were placed in ice-cold KB solution. The temperature of the perfusion was maintained at 37°C. Isolated cardiac myocytes were stored in KB solution at 4°C until needed [28].

### 9. Cellular electrophysiology recording

All experiments were carried out at room temperature (20–22°C). The whole cell clamp recording protocols, data acquisition and analysis were described in Methods S1.

### 10. Real-time PCR analysis

RNA levels of the Tgfβ1, Col1α1 and Col3α1 were determined by real-time PCR in Wild-type and Rgs5^−/−^ atria. The mRNA expression of Rgs5^−/−^ hearts was quantified relative to wild-type.

### 11. Statistical analysis

All data are expressed as mean ± SEM. Statistical analysis performed with a Fisher exact test and Student's t test were completed by SPSS 16.0. A value of P<0.05 was considered significant. Patch-clamp data were analyzed using Origin 6.0 (Microcal Co. USA) for nonlinear curve fitting.

## Results

### 1. ECG recording and echocardiography determination

**Figure 1:**
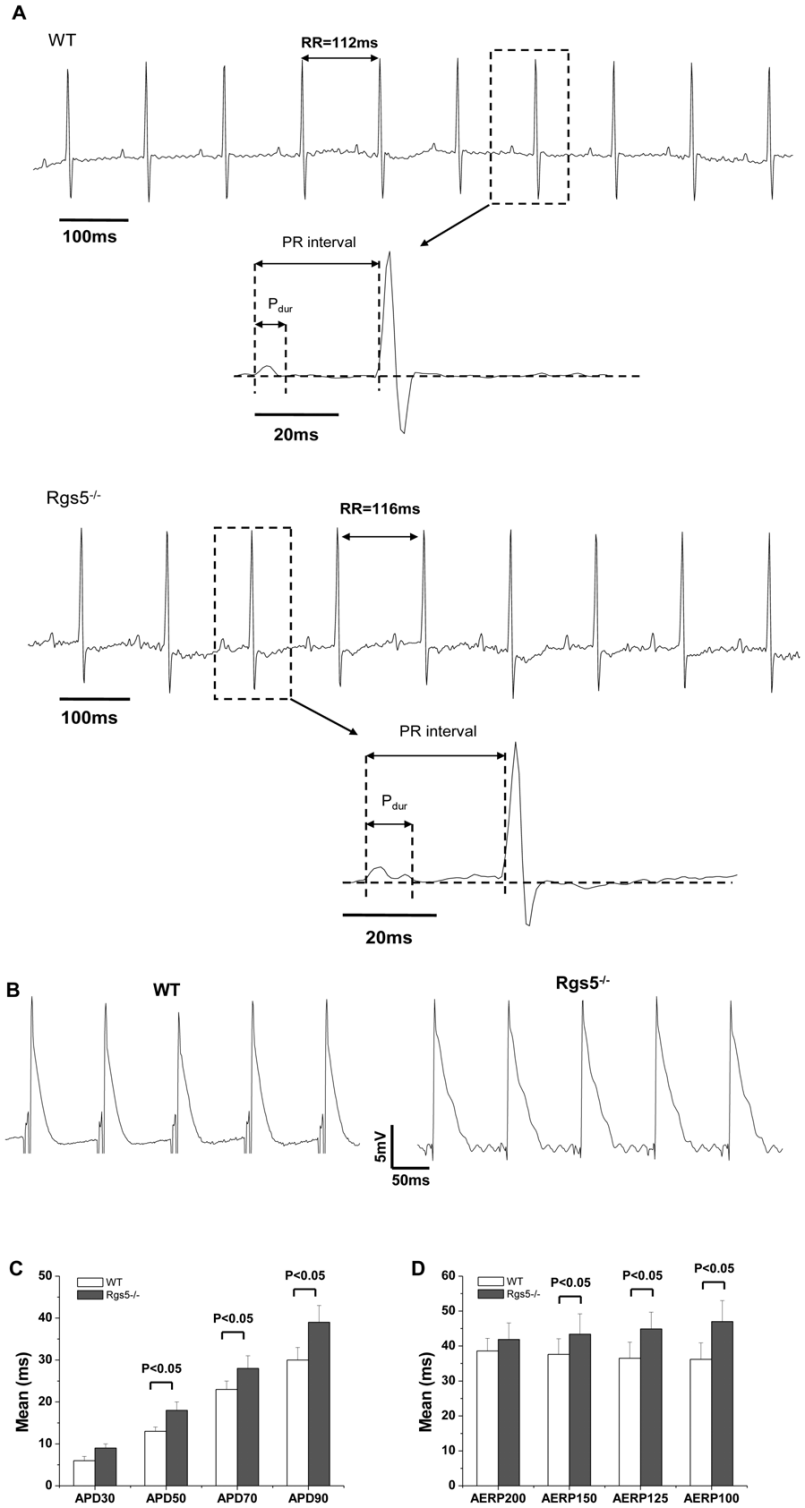
ECG and MAP recordings in Rgs5-/- and WT mice. Two representative electrocardiograms recorded from telemetry ECG showed the measurements of Pdur and PR intervals (A). Compared with wild-type mice, Rgs5^−/−^ showed widened P wave, and the action potential duration (APD) prolonged in the atria of Rgs5^−/−^ from 50% (APD_50_) to 90% (APD_90_) (B,C). The effective refractory periods (ERP) also significantly increased during S_1_ cycle length of PES at 150 ms, 125 ms and 100 ms.

To analyze whether Rgs5 deficiency results in atrial electrical abnormalities, telemetry ECG were performed. The spontaneous atrial arrhythmias were not observed during 24 h continuous recording, and there was no difference between Rgs5^−/−^ and wild-type mice in heart rate (HR) (P>0.05). However, the P wave usually showed widened in lead II of Rgs5^−/−^ mice, and its duration (P_dur_) was significantly different to that in wild-type mice (11.3±2.3 ms vs 9.4±2.4 ms, P<0.05) (Table 1 and Fig. 1). Echocardiography was applied to confirm whether this prolonged P wave is associated with atrial chamber dilation. The left atrial diameter was similar between WT and Rgs5^−/−^ mice (1.43±0.03 mm vs 1.40±0.08 mm, P>0.05). In addition, Rgs5^−/−^ mice did not exhibit ventricular dysfunction by determination of left ventricular ejection fraction (LVEF) and fractional shortening (FS) (Table 2).

**Table 1:** Measurement of ECG parameters between Rgs5^−/−^ and wild-type mice.

	*WT (n = 10)*	*Rgs5^−/−^ (n = 10)*	P
HR(bpm)	515.8±46.8	505.8±52.6	NS
RR(ms)	118.5±7.7	120.6±9.4	NS
PR(ms)	34.0±1.9	36.1±1.5	<0.05
P_dur_(ms)	7.3±1.1	10.6±2.5	<0.05
P_amp_(mV)	0.19±0.03	0.18±0.04	NS
QRS(ms)	8.0±0.1	8.1±0.4	<0.05

Heart rate (HR); RR interval (RR); PR interval (PR); P wave duration (P_dur_); P wave amplitude (P_amp_); QRS duration (QRS). NS: no statistical significance.

**Table 2:** Echocardiography parameters in WT and Rgs5^−/−^ mice.

	*WT (n = 8)*	*Rgs5^−/−^ (n = 8)*	P
HR (bpm)	458.7±39.7	449.0±31.9	NS
LVEDD (mm)	3.60±0.13	3.51±0.12	NS
LVESD (mm)	2.08±0.02	2.09±0.04	NS
LAD (mm)	1.43±0.03	1.40±0.08	NS
LVEF (%)	78.11±1.18	80.11±2.04	NS
FS (%)	43.66±1.45	41.35±2.81	NS

HR, heart rate; LVEDD, left ventricular end-diastolic diameter; LVESD, left ventricular end-systolic diameter; LAD, left atrial diameter, LVEF, left ventricular ejection fraction; FS, fractional shortening. NS: no statistical significance.

### 2. Rgs5^−/−^ mice have increased the duration of atrial repolarization

Monophasic action potentials (MAP) were obtained from isolated hearts with amplitudes ranging from 4.8 to 15.1 mV; the mean values did not significantly differ between the two groups. MAP duration (APD) from 30% to 90% was longer in Rgs5^−/−^ than WT mice during regular atrial pacing (CL = 125 ms) (Fig. 1). Similarly, PES revealed longer AERP in Rgs5^−/−^ mice than in wild-type mice, with significant differences during S1 paced CL at 150 ms (43.4±8.8 ms vs 37.6±6.5 ms, P = 0.04), 125 ms (44.9±8.8 ms vs 36.5±6.6 ms, P<0.01) and 100 ms (47.0±11.1 ms vs 36.2±4.7 ms, P<0.01) (Fig. 1 and Table 3).

**Figure 2:**
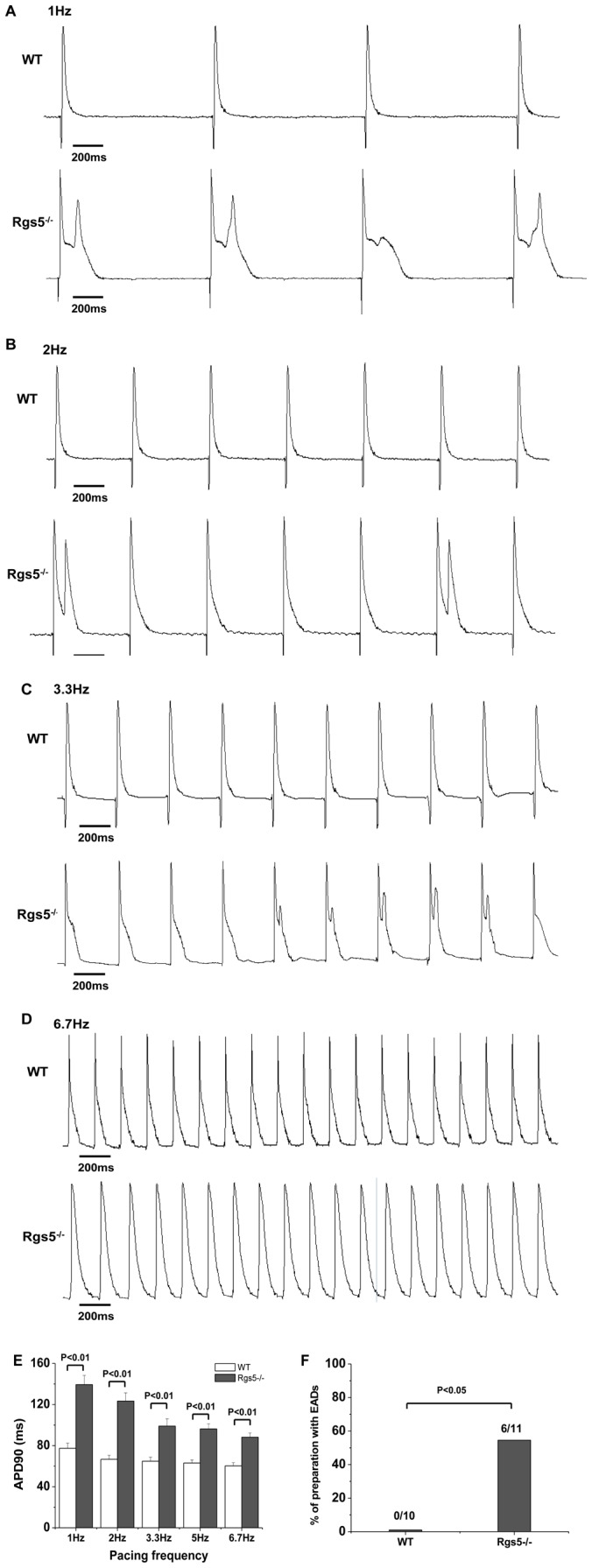
Transmembrane action potentials (TAP) from WT and Rgs5^−/−^ atrial samples. The TAP was recorded at paced frequency from 1 Hz to 6.7 Hz (A–D). Mean±SEM APD values at 90% repolarization (APD_90_) show APD prolongation at different pacing frequency (E). Early after-depolarizations (EADs) occurred in Rgs5^−/−^ group at 1 Hz, 2 Hz and 3.3 Hz. EADs occurrence was quantified as percentage of preparations (F).

Transmembrane action potential (TAP) from Rgs5^−/−^ and WT isolated sample of atrium was recorded at different paced CL due to very little spontaneous automaticity. Consistent with MAP findings, TAP recordings at Rgs5^−/−^ samples showed increased APD_90_ compared to WT group over all paced frequency from 1 Hz to 6.7 Hz. After-depolarization such as EAD was occurred frequently at 1 Hz and 2 Hz in Rgs5^−/−^ group. Moreover, EAD developed in 6 of 11 atrial preparations (54.5%), but none of 10 WT preparations (P<0.05) (Fig. 2).

**Table 3:** Electrophysiological parameters recorded in isolated mouse heart.

	*WT (n = 18)*	*Rgs5^−/−^ (n = 18)*	P
RR	154.6±28.3	167.5±32.1	NS
AERP_200_	38.6±7.6	41.9±7.7	NS
AERP_150_	37.6±6.5	43.4±8.8	<0.05
AERP_125_	36.5±6.6	44.9±8.8	<0.01
AERP_100_	36.2±4.7	47.0±11.1	<0.01
AVERP_200_	50.0±12.0	58.2±16.3	NS
AVERP_150_	53.0±11.2	59.0±17.3	NS
AVERP_125_	51.9±12.8	54.9±13.6	NS
AVERP_100_	52.6±14.2	46.2±6.5	NS
WCL	66.8±11.9	87.1±20.6	<0.01
SNRT	237.5±45.5	312.4±67.9	<0.01
cSNRT	84.9±11.2	144.8±31.7	<0.01
SNRTi	1.5±0.1	1.8±0.4	<0.01

Values are means±SE. AERP, atrial effective refractory periods; AVERP, atrioventricular effective refractory periods; WCL, Wenkebach cycle length. SNRT, sinus nodal recovery time; cSNRT, corrected sinus nodal recovery time; SNRTi, sinus nodal recovery time index. Subscripts 200, 150, 125 and 100 refer to S_1_ drive cycle lengths of 200, 150,125 and 100 ms, respectively. NS: no statistical significance.

### 3. Dysfunction of sinus and atrioventricular node in Rgs5^−/−^ mice

**Figure 3:**
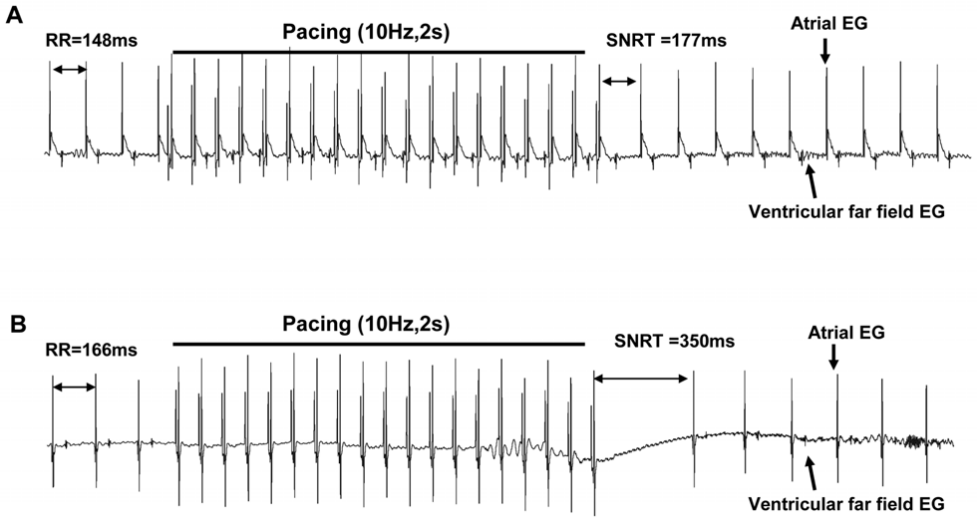
Sinus node recovery time (SNRT) in WT (A) and Rgs5^−/−^ (B) heart. The SNRT was measured by a 2 s pacing train at a cycle length of 100 ms, and defined as the interval between end of the stimuli and recovered sinus rhythm. The bipolar recording electrode was placed on the atrial appendage.

Sinus node automaticity was assessed by analyzing the SNRT. Atrial pacing confirmed sinus node dysfunction in Rgs5^−/−^ mice, with SNRT, corrected SNRT and SNRT index being longer in Rgs5^−/−^ hearts than in wild-type hearts (SNRT 312.4±67.9 ms vs 237.5±45.5 ms, P<0.01; cSNRT 144.8±31.7 ms vs 84.9±11.2 ms, P<0.01; 1.8±0.4 vs 1.5±0.1, P<0.01) (Table 3 and Fig. 3). In addition, AVERP was recorded in each S1 paced CL, but there were no significant differences between Rgs5^−/−^ and wild-type mice. However, the wenckebach cycle length (WCL) was significantly longer in Rgs5^−/−^ mice than in wild-type mice (87.1±20.6 ms vs 66.8±11.9 ms, P<0.01) by decremental atrial pacing (Table 3).

### 4. Rgs5^−/−^ heart increased the incidence and duration of atrial tachyarrhythmia

**Figure 4:**
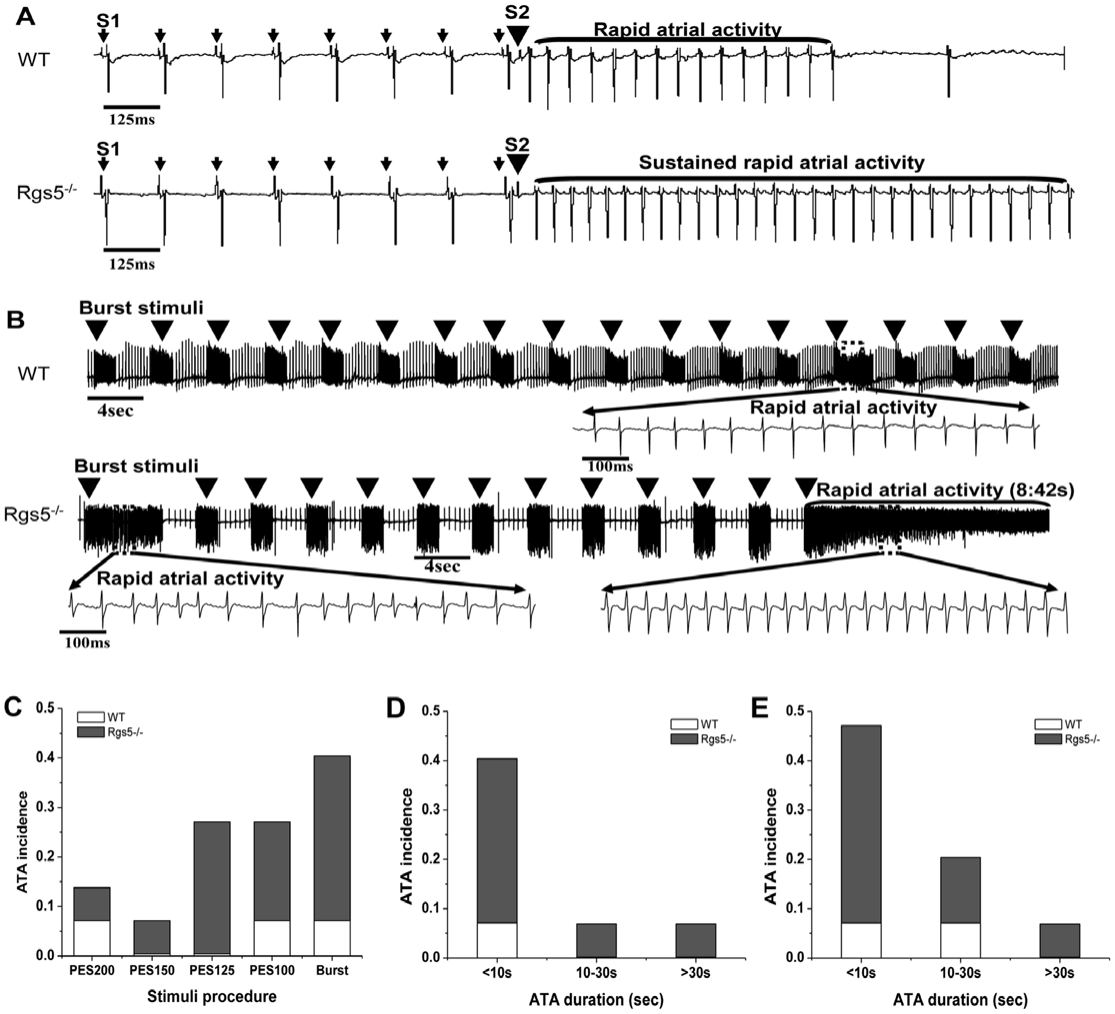
Atrial tachyarrhythmia (ATA) inducibility in Rgs5^−/−^ and WT hearts. PES (A) and Burst stimuli (50 Hz, 2 s) (B) were applied to induce atrial tachyarrhythmia (ATA). The sustained rapid atrial activities (duration>30 s) were elicited in Rgs5^−/−^ hearts, but Wild-type hearts showed non-sustained rapid atrial activities during both procedures. Overall incidence of ATA induced by PES and burst pacing in WT and Rgs5^−/−^ hearts (C), and the incidence was sorted by the ATA duration (<10 s, 10–30 s and >30 s) during PES (D) and burst pacing (E).

Atrial PES and burst pacing were applied to assess the arrhythmic tendency in Rgs5^−/−^ (n = 15) and wild-type (n = 14) hearts. Recordings were obtained during PES at a stimulation frequency from 5 Hz to 10 Hz. The S_2_ stimulus was continued until atrial refractoriness or arrhythmia occurred. A burst pacing procedure then applied high-frequency trains of S_1_ stimuli to the right atrium. Each heart reverted to sinus rhythm after arrhythmia. The Rgs5^−/−^ group showed a higher overall incidence of atrial tachyarrhythmia than the WT group through the procedures (P<0.05) (Fig. 4C).

The individual episodes of atrial arrhythmia were sorted by duration within the ranges <10 s, 10–30 s, and >30 s. Most ATA lasted <10 s in both groups, but the incidence of <10 s ATA was significantly higher in the Rgs5^−/−^ group than in the WT (P<0.05). Sustained ATA (>30 s) was induced in one Rgs5^−/−^ heart in both stimulation procedures. In contrast, arrhythmia lasting >30 s was not recorded in WT hearts during either PES or burst pacing (Fig. 4D,E and Table 4).

**Table 4:** Incidence of atrial tachyarrhythmia induced by PES and burst pacing.

	WT (n = 14)	Rgs5^−/−^ (n = 15)
**Inducibility**		
PES_200_	7.1% (1/14)	6.7% (1/15)
PES_150_	0	6.7% (1/15)
PES_125_	0	26.7% (4/15)^*^
PES_100_	7.1% (1/14)	20.0% (3/15)^*^
Burst	7.1% (1/14)	26.7% (4/15)^*^
**PES Duration**		
<10 s	7.1% (1/14)	33.3% (5/15)^*^
10–30 s	0	6.7% (1/15)
>30 s	0	6.7% (1/15)
**Burst Duration**		
<10 s	7.1% (1/14)	40.0% (6/15) ^*^
10–30 s	7.1% (1/14)	13.3% (2/15) ^*^
>30 s	0	6.7% (1/15)

Subscripts 200, 150, 125 and 100 refer to S_1_ drive cycle lengths of 200, 150,125 and 100 ms, respectively. * P<0.05 Rgs5^−/−^ vs WT.

### 5. K^+^ current remodeling in Rgs5^−/−^ atrial cardiomyocytes

**Figure 5:**
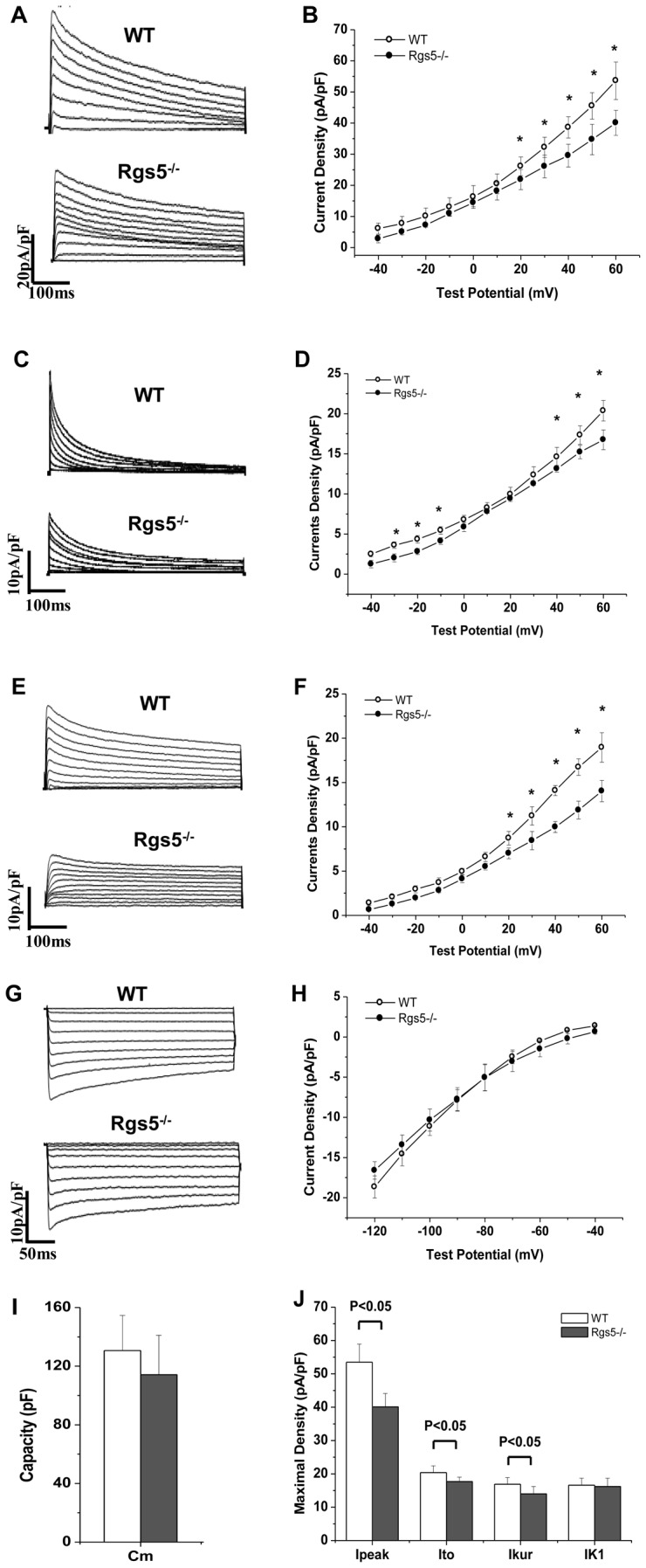
Alterations in K^+^ current were recorded in atrial myocytes of Rgs5^−/−^ and WT mice. Representative whole-cell total outward K^+^ currents (I_peak_) (A,B), transient outward currents (Ito) (C, D) and ultra-rapid delayed rectifier currents (I_kur_) (E, F) evoked during 500 ms test potential steps from −40 mV to +60 mV from a holding potential (HP) of −80 mV. The inwardly rectified K^+^ currents (I_K1_) (G, H), evoked during 350 ms voltage steps to potentials between −120 and −40 mV (HP = −80 mV). The current-voltage relationship (I–V) of I_peak_ and I_K1_ were plotted (B, D). Membrane capacity (C_m_) (I) and maximal densities of currents (J) were compared between wild-type and Rgs5^−/−^ mice. *P<0.05 WT vs Rgs5^−/−^.

**Figure 6:**
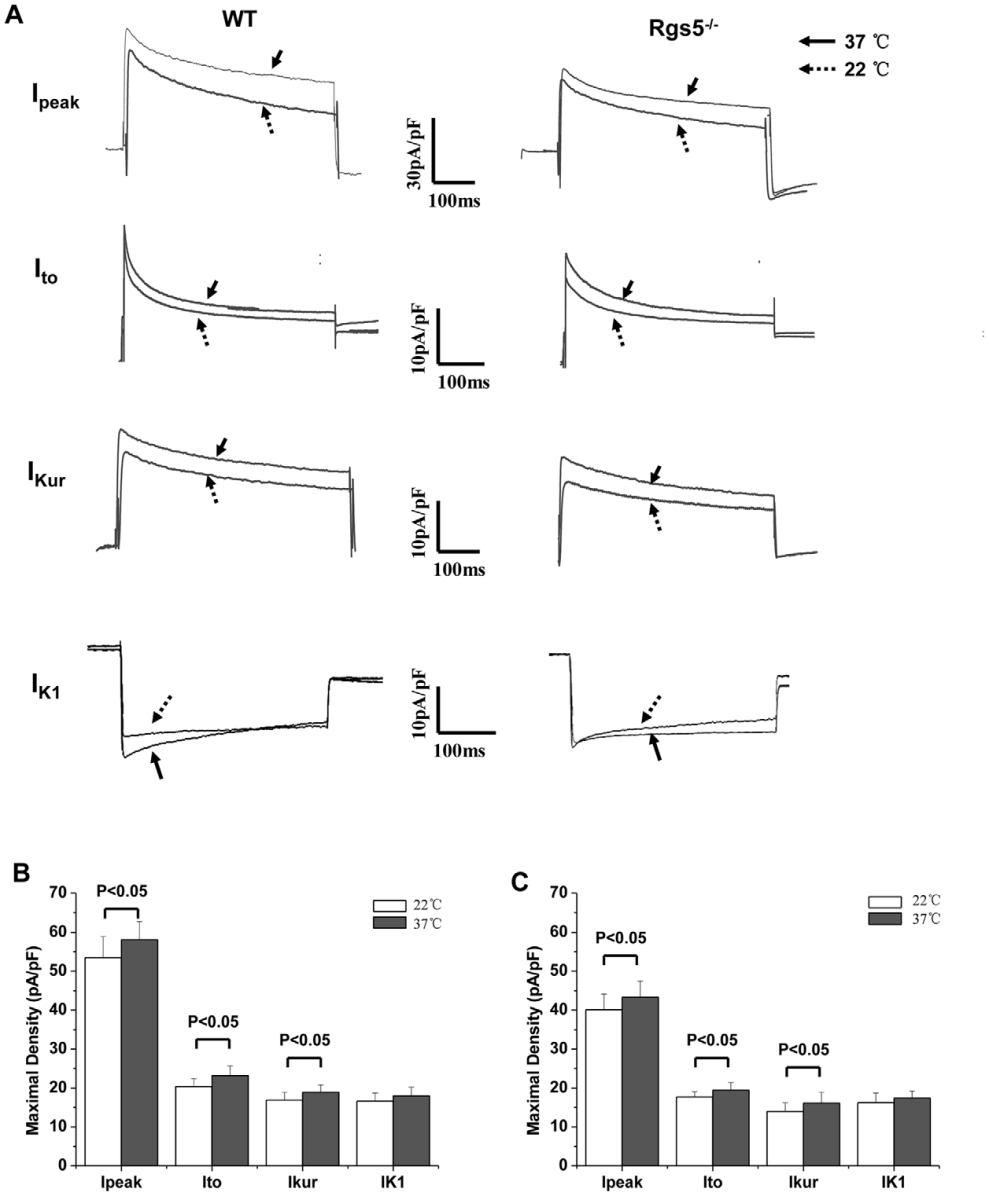
Comparison of magnitude of I_peak_, I_to_, I_Kur_ and I_K1_ in atrial myocytes of WT and Rgs5^−/−^ mice recorded at 22°C and 37°C (A). Bar graph represents mean (±S.E.M) maximal current density of each of the four outward K^+^ current recorded at 22°C and 37°C in WT (B) and Rgs5^−/−^ (C) group.

Whole-cell recordings were performed with a mean resting membrane potential of-77.6±2.1 mV and 78.3±1.8 mV in WT and Rgs5^−/−^ group, respectively (P>0.05). And the mean capacitance of each group was similar (WT: 130.6±23.9 pF; Rgs5^−/−^: 114.2±26.8 pF, P>0.05). The results revealed that the density of outward K^+^ current was markedly changed in Rgs5^−/−^ cardiomyocytes at room temperature (22°C). The I–V curves of total outward current (I_Peak_) showed the current density at +60 mV was decreased in Rgs5^−/−^ atrial cells compared to wild-type cells (53.5±5.4 pA/pF vs 40.1±4.0 pA/pF, P<0.05) (Fig. 5A). The two components of the I_Peak_, the transient outward (I_to_) and the ultra-rapid delayed rectifier (I_kur_), revealed significantly lower density in Rgs5^−/−^ cardiomyocytes than in the wild type (I_to_ at +60 mV: 16.7±1.3 pA/pF vs 20.4±2.0 pA/pF, P<0.05; I_kur_ at +60 mV: 14.0±2.2 pF/pA vs 17.9±2.0 pF/pA, P<0.05) (Fig. 5C,E). In addition, the I–V curves of inwardly rectified K^+^ current (I_K1_) showed similar densities at −120 mV in Rgs5^−/−^ and wild-type cardiomyocytes (−16.6±2.5 pA/pF vs −18.6±2.1 pA/pF, P>0.05) (Fig. 5G). To determine whether this results are also convinced when data are obtained at more physiological temperatures, we performed the measurement at 37°C. As expected, the mean maximal density of I_peak_, I_to_ and I_Kur_ was significantly larger at 37°C than at room temperature (P<0.05) (Fig. 6). The difference of density between Rgs5^−/−^ and WT also revealed significantly at 37°C in I_peak_, (at +60 mV: 44.3±4.2 pA/pF vs 59.2±4.7 pA/pF, P<0.05), I_to_ (at +60 mV: 20.4±2.0 pA/pF vs 23.2±2.5 pA/pF, P<0.05) and I_Kur_ (at +60 mV: 17.1±1.5 pF/pA vs 19.2±1.9 pF/pA, P<0.05).

**Figure 7:**
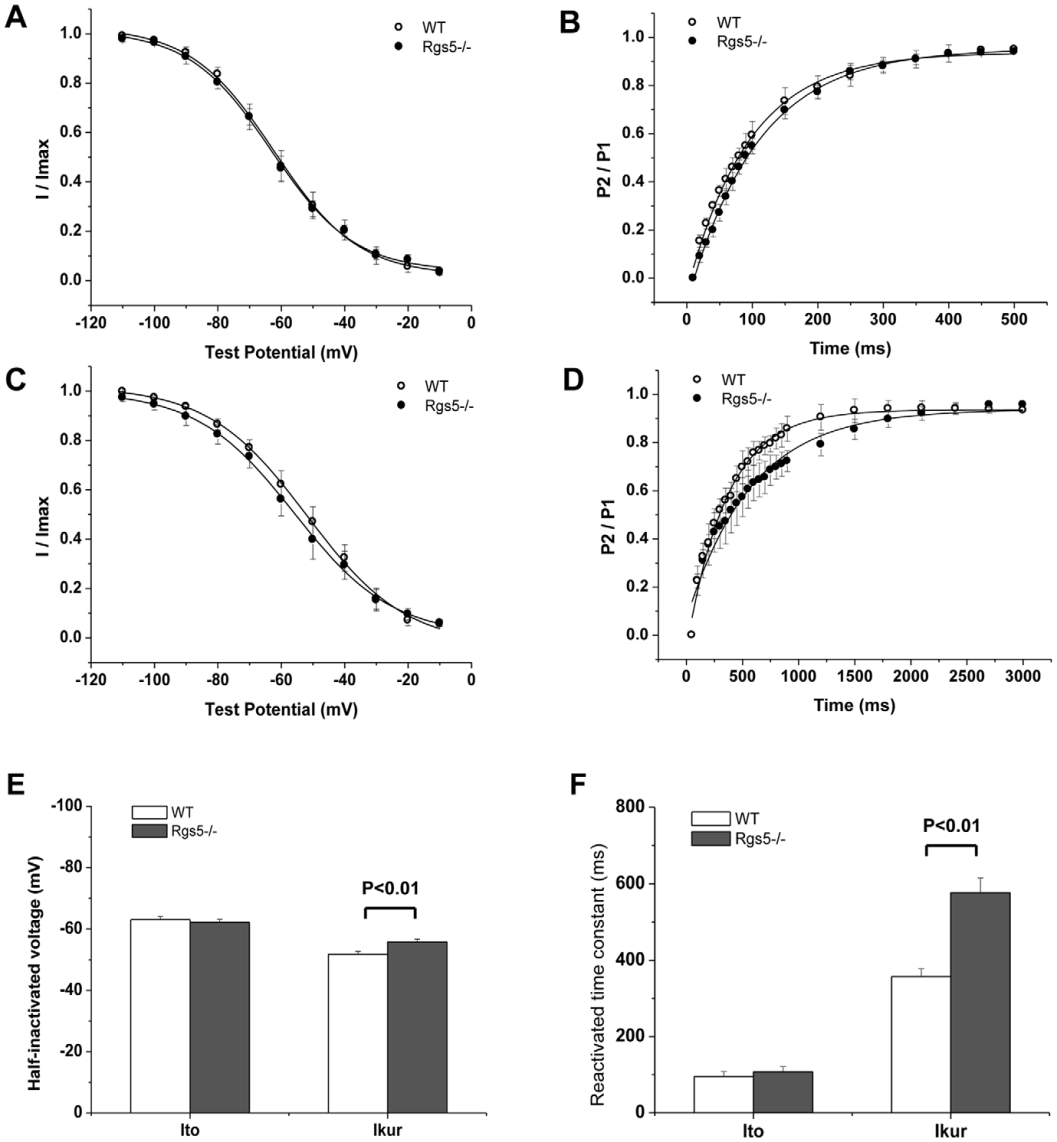
Channel kinetics of I_to_ and I_Kur_ were analyzed in Rgs5^−/−^ and WT cardiomyocytes, respectively. The steady-state inactivation (A) and reactivation (B) of curves in I_to_ plotted similar tendency between Rgs5^−/−^ and wild-type atrial myocytes. However, the property of I_Kur_ in Rgs5^−/−^ cardiomyocytes showed markedly modification with significant difference of half-inactivated voltage (V_1/2_) (C) and reactivated time constant (τ) (D). Histogram summarized the statistical significance of V_1/2_ and τ in WT and Rgs5^−/−^ groups (E, F).

To analyze the kinetics of I_to_ and I_kur_, the properties of inactivation and recovery were assayed in each group (Fig. 7). For the voltage dependence of steady-state inactivation of I_to_, the half-inactivated voltage (V_1/2_) and slope factor (k) showed no significant difference between Rgs5^−/−^ and wild-type cells (V_1/2_: −63.1±1.0 mV vs −62.2±1.0 mV, P = 0.13; k: 13.0±0.8 mV vs 13.2±0.7 mV, P P>0.05). However, the recovery from inactivation of I_to_ was significantly slower in Rgs5^−/−^ myocytes than in the wild type (time constant: 107.5±13.6 ms vs 95.1±13.2 ms, P>0.05). For I_kur_, the V_1/2_ (−51.8±0.9 mV vs −55.8±0.9 mV, P<0.01) and the recovery time (356.9±20.6 ms vs 577.0±38.3 ms, P<0.01) were significantly different between WT and Rgs5^−/−^ cardiomyocytes.

### 6. Effect of Rgs5^−/−^ on atrial fibrosis

**Figure 8:**
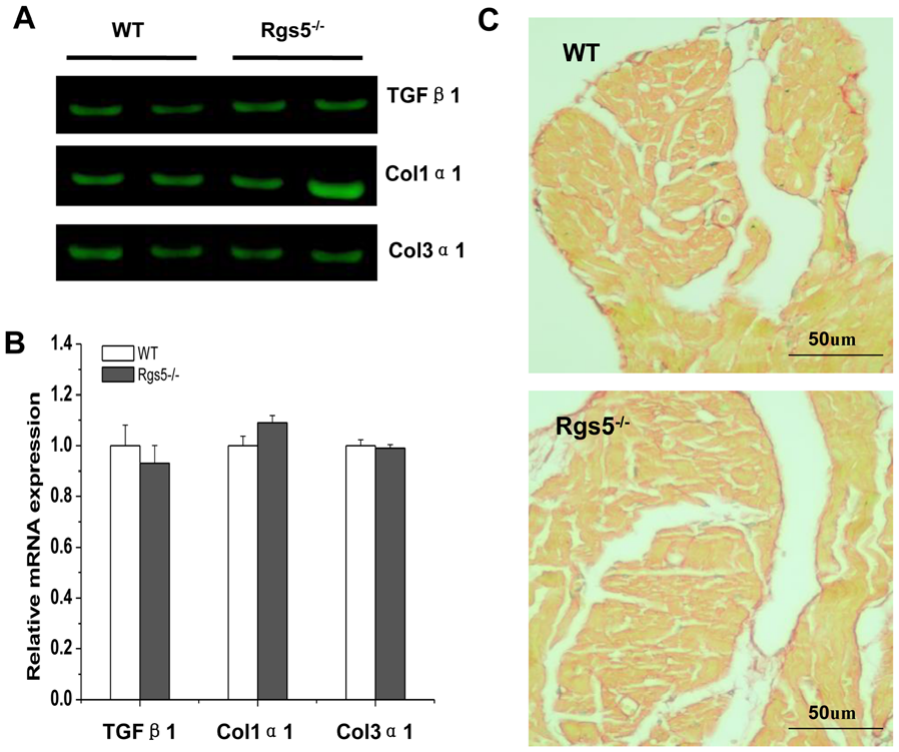
Fibrosis assay in Rgs5^−/−^ and WT atrium. mRNA expression levels of mediators of fibrosis including Tgfβ1, procollagen, type Iα1 (Col1α1) and procollagen, type IIIα1 (Col3α1) were analysis in WT and Rgs5^−/−^ mouse atrium (A, B). Relative abundance was calculated with the value of wild-type as a reference of 100%. PSR staining on histological sections of the atrial appendage and ventricle was performed in WT and Rgs5^−/−^ mice (C).

The analysis of mRNA expression levels of mediators of fibrosis including Tgfβ1, procollagen, type Iα1 (Col1α1) and procollagen, type IIIα1 (Col3α1) revealed a similar tendency between WT and Rgs5^−/−^ mouse atrium (P>0.05) (Fig. 8A,B), suggesting that the Rgs5^−/−^ did not promote the formation of fibrosis in atrium. PSR staining section confirms that morphologically through calculated the relative area of fibrosis from atria of WT (n = 8) and Rgs5^−/−^ (n = 8), the area occupied by fibrosis was 12.1%±3.4% in WT and 10.9%±2.7% in Rgs5^−/−^, it showed not significantly difference between two groups (P>0.05) (Fig. 8C).

## Discussion

### 1. Main findings

The present study provided an important original insight into the atrial electrical remodeling of Rgs5^−/−^ mice, As the consequence, we found that (i) Rgs5^−/−^ significantly prolonged atrial repolarization associated with attenuated K^+^ currents; (ii) the increased incidence and duration of atrial arrhythmia in Rgs5^−/−^ mice may related to the prolonged repolarization.

### 2. Rgs5 and atrial repolarization

G proteins are key mediators of cardiac signal transduction, particularly following activation of G protein coupled receptor (GPCR). G proteins include α (Gα) and βγ (Gβγ) subunits. Different types of Gα can be activated by special receptors [18]. For example, Gαi can be activated by muscarinic cholinergic receptors, Gαs by β-adrenergic receptors, and Gαq by angiotensin II type 1 receptor (AT1R) and endothelin-1 receptor A (ET_A_). RGS5, a member of the regulator of G-protein signaling protein super-family, acts as a GTPase-activating factor for a number of Gαi- and Gαq-mediated pathways and as a negative regulator of GPCR signaling [5]. Our previous study demonstrated that Rgs5 transgenic mice were resistant to cardiac hypertrophy and fibrosis through inhibition of MEK-ERK1/2 signaling, whereas Rgs5^−/−^ mice displayed the opposite phenotype in response to pressure overload [15]. Knockdown of RGS5 results in the selective up-regulation of AT1R-mediated activation of ERK1/2 [17]. Previous studies have demonstrated a relationship between AT1R and K^+^ ion in several tissues. AngII can inhibit several K^+^ currents, including K_v_, K_ATP_, and B_Kca_, and its effects on K^+^ currents can be reversed by blocking AT1R [9], [11], [23], [25]–[26]. Importantly, AT1R over-expressing mouse hearts had delayed repolarization and reduced density of several repolarizing K^+^ currents, together with an increased incidence of spontaneous arrhythmia [14]. Thus, as a negative mediator of GPCR signaling pathways, Rgs5 may directly regulate atrial repolarization through K^+^ currents.

As the primary repolarizing component in mouse heart, the voltage dependent K^+^ current exhibits more powerful than other mammals [8]. Decreased K^+^ currents may seriously prolong repolarizing phase. In present study, we observed the atrial action potential duration was prolonged in Rgs5^−/−^ mice, which associated with greatly decreased K^+^ currents including I_Peak_, I_to_ and I_Kur_, but the inwardly rectified K^+^ current (I_K1_) did not showed significant difference between Rgs5^−/−^ and WT group. Rivard et al. demonstrated that prolongation of APD in AT1R-overexpressing mouse hearts was due to decreased I_to_ and I_Kur_ densities and alteration of their reactivation kinetics by chronic stimulation of the AT1R signaling pathway. Notably, these alterations preceded anatomic disturbances and were not a consequence of cardiac remodeling [20]. However, the reduced density of the I_K1_ current is more common in mouse heart with cardiac dysfunction and increased myocytes size [3]. In present study, Rgs5^−/−^ heart did not show increased atrial size and cellular membrane capacitance compared to WT mice, and the function of Rgs5^−/−^ heart was normal. Based on these observations, it has been concluded that reductions of I_K1_ are closely related to cardiomyocyte hypertrophy and may not be directly due to the increased stimulation of AT1R. Consistent with these notions, the markedly decreased densities of I_Kur_ and I_to_ in Rgs5^−/−^ atria possibly contributed to the effect on the AT1R/Gq signaling pathway.

### 3. Rgs5 and atrial tachyarrhythmia

Toumi et al reported that Rgs2, another member of the RGS protein super-family, is expressed at high levels in mouse atrium and plays a critical role in atrial arrhythmias. Rgs2^−/−^ mice were more susceptible to PES-induced atrial tachycardia and fibrillation (AT/AF), and a higher percentage of them were accompanied by heterogeneities in refractory periods and regional conduction block. This phenotype was likely due to the reentry mechanism [27]. Atrial tachyarrhythmia can be initialized by rapidly firing ectopic foci (e.g., from the pulmonary veins) and maintained by functional reentrant circuit (mother rotor) [12], [19]. As a major risk factor for atrial arrhythmogenesis, prolonged repolarization forms the substrate for ectopic stimulation, which is generated from pulmonary and caval vein, cardiac ganglion or other atrial special tissues. Several studies have examined predisposition to atrial arrhythmia in mouse models of congenital long QT syndrome (LQT). Dautova et al. showed that in mice with a ΔKPQ knock-in LQT3 mutation, Langendorff-perfused hearts are more likely to develop atrial arrhythmia [13]. Lemoine et al. demonstrated that atrial arrhythmias induced by rapid rate alteration occurred significantly more often in the LQT3 mouse model, suggestive of after-depolarization-dependent arrhythmogenic mechanisms [21]. In present study, although we did not observe the spontaneous atrial arrhythmia during 24 h-ECG recording, the trigger activity including EAD was observed in Rgs5^−/−^ atrial tissue, and the electrical stimulation (PES and burst pacing), which simulate a physiological ectopic excitation and is thought to more readily induce reentry than triggered activity [27], induced atrial arrhythmias were facilitated in Rgs5^−/−^ heart. It suggested that Rgs5^−/−^ induced prolonged repolarizaiton provided a potential substrate and the ectopic activity facilitated this effect for arrhythmogenesis in atria.

As the substrate for atrial arrhythmogenesis, interstitial fibrosis and atrial enlargement have been implicated in theoretical models. In present study, we did not observed significant sign of atrial enlarged or fibrosis. However, the prolonged P waves were recorded in Rgs5^−/−^ conscious mice. As a sign of atrial enlargement, prolonged P wave was also seemed to be related to regional atrial conduction disturbances, which have been suggested to favour the development of AF [29]. Thus, the result suggested that the Rgs5^−/−^ induced ATA was mainly dependent on the atrial electrophysiological modifications.

In addition, SNRT was measured to assess the function of the sinus node, which maintains the normal sinus rate. Several RGS mRNAs are expressed at high levels in the sinus node and atrioventricular nodal region and regulate parasympathetic tone of pacemaker myocytes. Rgs4 and Rgs6 are highly expressed in the sinus node, and hearts from mice deficient in these genes showed marked bradycardia with a sustained lower HR and prolonged RR intervals [6], [10], [22]. Alterations in RGS protein expression or activity could potentially contribute to diseases such as sick sinus syndrome. The Rgs5^−/−^ hearts, which have significantly longer SNRT and cSNRT, had abnormal sinus function and thus promoted the maintenance of atrial ectopic rhythm.

### Conclusion

In conclusion, the present study confirmed the relationship between the Rgs5 and atrial arrhythmogenesis, the Rgs5^−/−^ facilitated ATA was due to the prolonged repolarization, and this electrophysiological alteration could be accounted for attenuated repolarizing K^+^ currents.

## Supporting Information

Methods S1 Supplemental Methods (DOC)
