# Supplementary material for: Atrial Tachyarrhythmia in Rgs5-Null Mice
Source: PLoS One. 2012 Nov 5;7(11):e46856. doi: 10.1371/journal.pone.0046856 (PMC3489853; doi:10.1371/journal.pone.0046856)
Supplement: Methods S1 Supplemental Methods — (DOC) [file pone.0046856.s001.doc]

**Supplement method**

**Experiment animals**

The generation and genotyping of Rgs5^-/-^ mice (C57BL/6 background) have been described previously. Mice were provided with food and water and held on standard 12 hours light and dark cycles in temperature and humidity controlled house. All protocols were conform to the Guide for the Care and Use of Laboratory Animals published by US National Institutes of Health (NIH Publication No.85-23, revised 1996), and approved by the Animal Care and Use Committee of Renmin Hospital of Wuhan University. Male Wild-type and Rgs5^-/-^ mice aged 8 to 10 weeks were used in the studies.

**Telemetry ECG recording**

Anesthetized (pentobarbital sodium 60mg/kg,i.p.) mice were positioned on a thermally controlled heating pad, body temperature was maintained at 37°C. Leads were tunneled subcutaneously to the right shoulder and left apex (lead II), respectively. Recordings began after mice recovered for >24 hours. The ECG amplifier module (DSI, US), which included high- and low pass filters (set to 0.05 Hz and 1kHz, respectively) and a gain selection device (set to 1000-fold). Signals were digitized continuously at 1 kHz and recorded by using the data acquisition system (DSI, US). The software (P3) was used to analyze the telemetry ECG recorded data. For each mouse, analysis of the cardiac rhythm and heart rate (HR) were performed on 24h continuous experimental recording. The P-wave duration and amplitude (P_dur_ and P_amp_), PR interval and QRS duration was measured.

**Transthoracic Echocardiography**

Echocardiography was performed by Sonos 5500 ultrasound (Philips) with a 15-MHz linear array ultrasound transducer. The LV was assessed in both parasternal long-axis and short-axis views at a frame rate of 120Hz. End-systole or end-diastole was defined as the phase in which the smallest or largest area of LV, respectively, was obtained. LVEDD, LVESD, ejection fraction (EF) and fractional shortening (FS) were measured from the LVM-mode tracing with a sweep speed of 50 mm/s at the midpapillary muscle level. Left atrial size was determined from long-axis two dimensional images at end-systole^1^.

**Histological analysis of fibrosis**

Hearts were excised, washed with saline solution, and then placed in 10% formalin. Hearts were cut transversely close to the apex to visualize the left and right ventricles and atrial appendages. Several sections of heart (4–5 μm thick) were prepared and stained with Picro Sirius Red (PSR) for collagen deposition and then visualized by light microscopy. Using Adobe Photoshop 7.0 software, the number of red (collagen) and yellow (tissue) pixels were analyzed and the percentage of fibrosis [red pixels/(red－yellow pixels)] was counted^2^.

**Preparation of Langendorff-perfused hearts**

After anesthesia with pentobarbital sodium (60mg/kg, i.p.) and heparinized by heparin sodium (100U, i.p.), the isolated heart was quickly excised and transferred to ice-cold (4°C) HEPES-buffered Tyrode’s solution (mM: NaCl 130; KCl 5.4; CaCl_2_ 1.8; MgCl_2_ 1; Na_2_HPO_4_ 0.3; HEPES 10; glucose 10; pH adjusted to 7.4 with NaOH), bubbled with 95% O_2_–5% CO_2_. The heart was then rapidly transferred and fixed to the langendorff-perfusion system (AD Instruments, Australia) after cannulated with a tailor-made 21-gauge cannula in aorta. Perfusion was commenced in a retrograde manner through the aorta at 2–2.5 ml/min by a peristaltic pump (AD Instruments, Australia). In this way, the heart was perfused by the HEPES-buffered Tyrode’s solution (37°C) passing through the aorta, into the coronary arteries. The isolated hearts were perfused for 20 minutes before further experimental test. The hearts that did not recover to the regular spontaneous rhythm or had inreversible myocardial ischemia were discarded^3^.

**MAP and BEG recording**

To examine the atrial electrical activity, the monophasic action potential (MAP) and bipolar electrogram (BEG) were recorded from the epicardial surface of atrial appendage using a custom-made electrode, constructed from two 0.25 mm Teflon-coated silver wire (99.99% purity), that were twist together and galvanically chlorided to eliminate DC offset. The paired platinum stimulating electrodes paced the epicardial surface of right atrial appendage and the stimulation used a 1ms square-wave stimuli at three times excitation threshold. These signals were amplified using an amplifier (AD Instruments, Australia) and band pass filtered between 0.05Hz and 1kHz. All digitized data was then captured and analyzed using Lab Chrat7.0 software.

**Transmembrane action potential (TAP) recording**

The samples of atrium were removed from heart and superfused with HEPES-buffered Tyrode’s solution (mM: NaCl 130; KCl 5.4; CaCl_2_ 1.8; MgCl_2_ 1; Na_2_HPO_4_ 0.3; HEPES 10; glucose 10; pH adjusted to 7.4 with NaOH), bubbled with 95% O2–5% CO2, at 37°C. TAP were recorded with borosilicate glass microelectrodes (resistance 10-20MΩ and filled with 3M KCl) and coupled to EPC-9 amplifier (List Instruments, Germany). The data was analyzed with Pulse-pulsefit software interface (Version 8.31, HEKA Co. Germany). Stimulation procedure was output with square wave pulses (2ms, 1.5×diastolic threshold) through bipolar Teflon-coated sliver electrodes. TAPs were recorded during regular pacing frequency of 1Hz, 2Hz, 3.3Hz, 5Hz and 6.7Hz.

**Electrical stimulated protocol**

The Programmed electrical stimulation (PES) protocol was used for atrial effective refractory period (AERP) and atrial-ventricular effective refractory period (AVERP) examinations. PES consisting of an eight stimuli (S1) drive train followed by a ninth extra-stimulus (S2), the CL of S1 train was under 200ms, 150ms, 125ms and 100ms, respectively. The first S1–S2 interval equalled the pacing interval and then progressively reduced by 1ms with each cycle until the S2 stimulus could no longer evoke an atrial deflection. Effective refractory period (AERP) defined as the longest S1–S2 interval that could not elicit an atrial deflection. Sinus node recovery time (SNRT) was measured after a 2s pacing train at a cycle length of 100ms, the definition was the interval between the last stimulus of the pacing train and the onset of the first sinus return beat, the SNRT defined as the interval between end of the stimuli and recovered sinus rhythm, the corrected SNRT (cSNRT) equaled to the values that SNRT subtracted the RR interval before stimulation^4^. Inducibility of atrial tachyarrhythmia was tested by using both PES and burst pacing (2 ms pulses at 50 Hz, 2s burst duration), burst pacing used up to 3 minutes of pacing in both atrial locations^5^.

**Isolation of atrial cardiac myocytes**

Rgs5^-/-^ and WT mice were heparinized (100U, i.p.) and anaesthetized by pentobarbital sodium (60mg/kg, i.p.). The hearts were removed and retrogradely perfused on Langendorff system with the following solutions: first, 5 minutes perfused with HEPES-buffered Tyrode’s solution (mM: NaCl 130; KCl 5.4; CaCl_2_ 1.8; MgCl_2_ 1; Na_2_HPO_4_ 0.3; HEPES 10; glucose 10; pH adjusted to 7.4 with NaOH). Second, 5 minutes perfused with Ca^2+^-free HEPES-buffered Tyrode’s solution. Third, the heart was perfused with Ca^2+^-free HEPES-buffered Tyrode’s containing 0.6mg/ml collagenase type II (Invitrogen Co. US), 0.1% bovine serum albumin, 20mM taurine and 30μM CaCl_2_ for 15min digestion. Fourth, at the end of digestion, KB solution (mM: taurine 10; glutamic acid 70; KCl 25; KH_2_PO_4_10; glucose 22; EGTA 0.5; pH adjusted to 7.2 with KOH) was applied to washout the remnant digested solution for 5min. The temperature of these perfusions was maintained at 37 °C. At the end of the perfusion, the atrial appendages were dissected from the heart and placed into KB solution. Then, the atrial appendages were chopped into several pieces and dispersed by gentle manual agitation using a 1mL tip transfer pipette. Isolated cardiac myocytes were stored in KB solution at 4°C until needed^6^.

**Cellular electrophysiology recording**

Whole-cell patch clamp was performed using EPC-9 amplifier (List Instruments, Germany) and data was recorded and analyzed with Pulse-pulsefit software interface (Version 8.31, HEKA Co. Germany). During the experiments, the myocytes were continuously superfused with the extracellular solution (2mL/min) containing (in mM): NaCl 130; KCl 5.4; CaCl_2_ 1; MgCl_2_ 1; Na_2_HPO_4_ 0.3; HEPES 10; glucose 10; (pH adjusted to 7.4 with NaOH). The resistances of the pipettes ranged from 2.5 to 3.5MΩ when filled with pipette solution (mM): K–aspartate 110, KCl 20, NaCl 8, MgC1_2_ 1, CaC1_2_ 1, MgATP 4, EGTA 0.1 and 10 HEPES (pH 7.2 with KOH). Series resistance (Rs) was between 4-8 MΩ and compensation was applied to reduce Rs by 80-90%. Current signals were filtered at 3 kHz by a 8-pole Bessel filter, digitized at a sampling rate of 1kHz, stored on the computer running Pulse-pulsefit software which was additionally used for the generation of voltage pulses and data analysis. All experiments were carried out at room temperature (20-22 °C).

K+ currents recordings: The total potassium (I_peak_) current-voltage relationship was elicited by a series of 500ms test potential steps from -40mV to +60mV with 10mV increments and a holding potential of -80mV at a frequency rate of 0.1 Hz. To examining outward potassium currents: Ito and I_Kur_, we first inactivated the transient outward currents (Ito) with applying pre-pulse (100ms, -40mV) before main activation steps. The density of Ito was obtained by compared the subtracted current traces with and without the inactivating pre-pulse. The remained current after pre-pulse is denoted I_Kur_. For inward rectified potassium currents (I_K1_), the 350ms steady-state depolarizing pulse from a holding potential of -80mV to step potentials between -140mV and -40mV. The amplitude of I_K1_ was measured by subtracted the background currents in myocytes before and after superfusion with 100uM BaCl_2_. Current densities (pA/pF) were obtained by normalizing the current amplitudes (pA) by the C_m_ (pF).

Steady-state inactivation: The two step voltage-clamp protocol is applied for steady-state inactivation of I_to_ and I_Kur_. It consisted of an inactivating pre-pulse varied from -110mV to -10mV with a period of 1s or 5s for I_to_ and I_Kur_, and followed by a fixed test pulse to +30mV during 1s (I_to_) or 5s (I_Kur_). For the I_Kur_ recording, a 100ms pre-pulse at -40mV was added before a 5s second pulse at +30mV to inactivated I_to_. The test current amplitude of I_to_ and I_Kur_ at each pre-pulse potential was normalized to the maximal amplitude of this current (I/I_max_). Data were best fitted to the Boltzmann formula.

Recovery from inactivation: the time dependence of reactivation firstly were measured using an inactivating pulse from -80mV to +30mV during period of 500ms or 1.5s for I_to_ and I_Kur_ , respectively. Then, a variable time intervals (10ms-500ms or 10ms-3000ms for I_to_ and I_Kur_ respectively) was followed a 500ms test pulse at +30mV. For I_Kur_ recording, an inactivating pulse (100ms at -40mV) was elicited between first inactivating and test pulse to inactivate I_to_. The ratio of the current amplitude elicited by the test pulse to the inactivating pulse (P2/P1) was plotted as a function of the intervals time. Time constant was calculated by data fitted to exponential functions.

**Real-time PCR assays**

Total RNA were extracted from frozen, pulverized mouse heart tissue using Trizol Reagent (Aidlab). cDNA fragments were synthesized by RT using the cloned reversed transcript kit (Aidlab). The primers were designed for Tgfβ1, Col1α1 and Col3α1 by using the Primer5.0 designing tool. The real-time PCR reaction was carried out with SYBR Green qPCR Mix (Aidlab) using a real-time PCR system (ABI stepone plus). The PCR reactions were cycled 40 times using a 3-step cycle procedure (denaturation at 95°C for 15s, annealing at 58°C for 20s, elongation at 72°C for 30s) after an initial stage at 95°C for 3 min to activate the Taq polymerase.

**Statistical analysis**

All data are expressed as mean ± SEM. Statistical analysis performed with a Fisher exact test and Student’s t test were completed by SPSS 16.0. A value of P<0.05 was considered significant. Patch-clamp data were analyzed using Origin 6.0 (Microcal Co. USA) for nonlinear curve fitting.

**Reference**

1. Li HL, He CW, Feng JH, Zhang Y, Tang QZ, et al. (2010) Regulator of G protein signaling 5 protects against cardiac hypertrophy and fibrosis during biomechanical stress of pressure overload. Proc. Natl. Acad. Sci. U.S.A 107: 13818–13823.
2. Stein M, Boulaksil M, Jansen JA, Herold E, Noorman M, et al. (2010) Reduction of fibrosis-related arrhythmias by chronic renin-angiotensin-aldosterone system inhibitors in an aged mouse model. Am J Physiol Heart Circ Physiol 299: H310–H321.
3. Ghais NS, Zhang Y, Mistry B, Grace AA, Huang CL. (2008) Anti-arrhythmic effects of cyclopiazonic acid in Langendorff-perfused murine hearts. Prog Biophys and Mol Bio 98: 281–288.
4. Mangoni ME, Traboulsie A, Leoni AL, Couette B, Marger L, et al. (2006) Bradycardia and slowing of the atrioventricular conduction in mice lacking CaV3.1/α1G T-Type Calcium Channels. Circ Res 98:1422-1430.
5. [Reil JC](http://www.ncbi.nlm.nih.gov/pubmed?term="Reil JC"%5BAuthor%5D), [Hohl M](http://www.ncbi.nlm.nih.gov/pubmed?term="Hohl M"%5BAuthor%5D), [Oberhofer M](http://www.ncbi.nlm.nih.gov/pubmed?term="Oberhofer M"%5BAuthor%5D), [Kazakov A](http://www.ncbi.nlm.nih.gov/pubmed?term="Kazakov A"%5BAuthor%5D), [Kaestner L](http://www.ncbi.nlm.nih.gov/pubmed?term="Kaestner L"%5BAuthor%5D),et al. (2010) Cardiac Rac1 overexpression in mice creates a substrate for atrial arrhythmias characterized by structural remodeling. Cardiovasc Res 87:485-93.
6. Brouillette J, Clark RB, Giles WR, Fiset C. (2004) Functional properties of K+ currents in adult mouse ventricular myocytes. J Physiol 559:777-798.
